# DNA repair in lupus

**DOI:** 10.1186/ar4623

**Published:** 2014-09-18

**Authors:** Jiadi Xu, Zubin Patel, Leah Kottyan, Richard A Gatti, Deborah K McCurdy, Kenneth M Kaufman, John B Harley

**Affiliations:** 1 Cincinnati Children's Hospital Medical Center, University of Cincinnati, Cincinnati, OH, USA; 2 University of California at Los Angeles, Los Angeles, CA, USA; 3 US Department of Veterans Affairs Medical Center, Cincinnati, OH, USA

## Background

The myriad of mouse genetic alterations that result in a lupus-like phenotype and the >100 genes now known to be involved in human lupus are all probably only a hint at the potential complexity of the many mechanisms that lead to systemic lupus erythematosus (SLE).

## Methods

We have applied exome sequencing to 24 SLE patients and their parents (trios) in an effort to conquer the data analysis and to identify candidate genes that may contribute when the activity of their gene products were substantially altered.

## Results

The proband of one of our SLE trios had *de novo *mutations in *RAD54B *that was predicted to change ARG to GLN and to be severely damaging to protein product activity by multiple algorithms. A second much more conservative *de novo *mutation in *DOCK8 *was not predicted to be consequential. *RAD54B* is a component of the homologous recombination DNA repair pathway. Cells from the SLE proband were unusually sensitive to ionizing radiation by the colony survival and comet tail assays. Ionizing radiation selectively induced interferon responsive genes in cells from this patient and not from controls. Transfection of the wild-type gene into the cells from this patient led to overexpression of the *RAD54B* gene product and returned ionizing radiation resistance toward normal.

## Conclusion

These data in addition to the other five other genes directly or indirectly involved in altering risk of SLE by influencing DNA repair (*TREX1, RAD51B, XRCC1, XRCC3*, and *XRCC4*) implicate base excision repair, nonhomologous end joining, and homologous recombination. These results suggest that multiple DNA repair mechanisms contribute to SLE susceptibility.

